# Trends of the contributions of biophysical (climate) and socioeconomic elements to regional heat islands

**DOI:** 10.1038/s41598-021-92271-3

**Published:** 2021-06-16

**Authors:** Shengzi Chen, Zhaowu Yu, Min Liu, Liangjun Da, Muhammad Faiz ul Hassan

**Affiliations:** 1 Shanghai Key Lab for Urban Ecological Processes and Eco-Restoration, School of Ecological and Environmental Sciences, East China Normal University, Shanghai, 200241 China; 2 Department of Environmental Science and Engineering, Fudan University, Shanghai, 200438 China; 3 School of Electronic Science and Engineering, University of Electronic Science and Technology, Chengdu, 610000 China

**Keywords:** Climate sciences, Ecology, Environmental sciences, Environmental social sciences

## Abstract

The development of urban accumulations in recent decades has led to the transformation of urban heat islands (UHI) into regional heat islands (RHI). The contributions of the biophysical, climate, and socioeconomic factors to RHI in urban agglomeration remain poorly understood. Here Yangtze River Delta urban agglomeration (YRDUA) in eastern China has been selected as a case area to explore the influences trends, of the influencing factors to RHI by using MODIS data from 2003 to 2017. Results showed that, in summer, the area fraction of daytime RHI in YRDUA has increased from 21.74 to 31.03% in 2003 and 2017, respectively. As compared to 2003, the annual nighttime RHI area in 2017 has increased from 7510 to 20,097 km^2^. The dominant factors of surface RHI intensity (SRHII) showed seasonal variation. Enhanced vegetation index (EVI) (interpretation of 33.27%) was the dominant factor of daytime SRHII in spring. The most important factor was normalized difference build-up density (NDBI) (37.28% and 26.83%, respectively) in summer and autumn. In winter, precipitation (26.16%) was the most influential. At night, Modified Normalized Difference Water Index (MNDWI) had a dominant effect on SRHII in spring (54.12%), autumn (52.62%), and winter (24.19%). The dominant factor of nighttime SRHII in summer was EVI (42%). Moreover, water bodies harm RHI during the day while having a positive effect at night. These findings can provide a theoretical basis for regional environment improvement and regional sustainable development.

## Introduction

Now, more than half of the world’s population lives in cities, and by 2050, the urban population will be more than double its current size^1^. Rapid urbanization has caused a series of urban environmental problems, including UHI, in which the temperature of the urban area is higher than that of the surrounding area^2^. UHI seriously affects air quality^3^, weather patterns^4–6^, energy consumption^7^, and human health^8^. With the development of urbanization, a new spatial organization form of urban development has emerged in recent decades: urban agglomeration. Urban accumulations are highly developed integrated urban spatial forms that are usually composed of more than one mega-city and more than three large cities within a specific area^9^. Unlike a single city, UHI in urban agglomerations is not a local phenomenon, and as the distance between cities decreases or disappears, urban agglomerations can greatly change the thermal environment of continuous regions^10^. Moreover, the interaction between heat islands in urban agglomerations may increase the UHI intensity or produce additive effects^11,12^, which may cause wide-ranging and more serious regional thermal environment problems.

Many previous studies have overestimated or underestimated UHI in urban agglomerations^13–15^, which may be caused by the diversity among the definitions of the UHI. For instance, UHI intensity was defined as the instantaneous or cumulative surface temperature contrast of the urban or core area of the city and the surrounding villages^16–18^, suburbs^13,19^, farmland^18^, forest^20^, water^21^. However, these definitions have two shortcomings. First, they assume that the suburbs or rural areas around a city do not produce a UHI, which leads to underestimations of the intensity of the UHI in the core cities^22^ and fails to reveal the specific model of heat island intensity in urban agglomerations. Moreover, the definitions of “city”^5,23^, “suburb”^24^, and “village”^25,26^ are ambiguous, which may delay assessments of the UHI in urban–rural assimilation areas or areas where the distance is shortened or even disappeared between cities^27^. Hence, it is essential to establish the evaluation method of UHI intensity in urban agglomeration.

Many factors can affect UHI, and they can be divided into three categories. (1) Biophysical factors, including land cover, vegetation, building density, and water bodies, with land cover change representing one of the most important reasons for the formation of RHI^28–30^ because the replacement of natural surfaces with impervious surfaces increases the sensible heat absorbed by the ground, and the heat cannot spread, which causes high temperatures in urban areas^16,17,31^. Many studies have shown that green vegetation^30^, building density^32^, and water bodies^33,34^ have a certain correlation with UHI. (2) Socioeconomic factors, which include the economic development level, population density (PD), and anthropogenic heat emissions. Most of the changes in a natural environment are related to human activities. (3) Climatic factors, which include air temperature (AT) and precipitation (PRE). AT has the most direct effect on UHI^4^, and PRE intensity has a mitigation effect on local heat islands^34^. Many studies found that there is seasonal variation between UHI and its drivers. For example, Peng et al.^13^ found that nighttime UHI intensity correlates directly with albedo and nighttime light (NL), while daytime UHI intensity correlates inversely with vegetation cover. Zhou et al.^19^ showed that UHI was strongest in summer and weakest in winter during the day. Zhang^35^ studied the temporal and spatial variation characteristics of UHI in Shanghai from 1978 to 2007. The results show that the UHI in Shanghai has been significantly increased from 1978 to 2007 with the strongest UHI in autumn and the weakest in summer. However, to point out the dominant influencing factor, changes in this factor with seasonal and inter-annual variability, require additional investigation.

**Figure 1:**
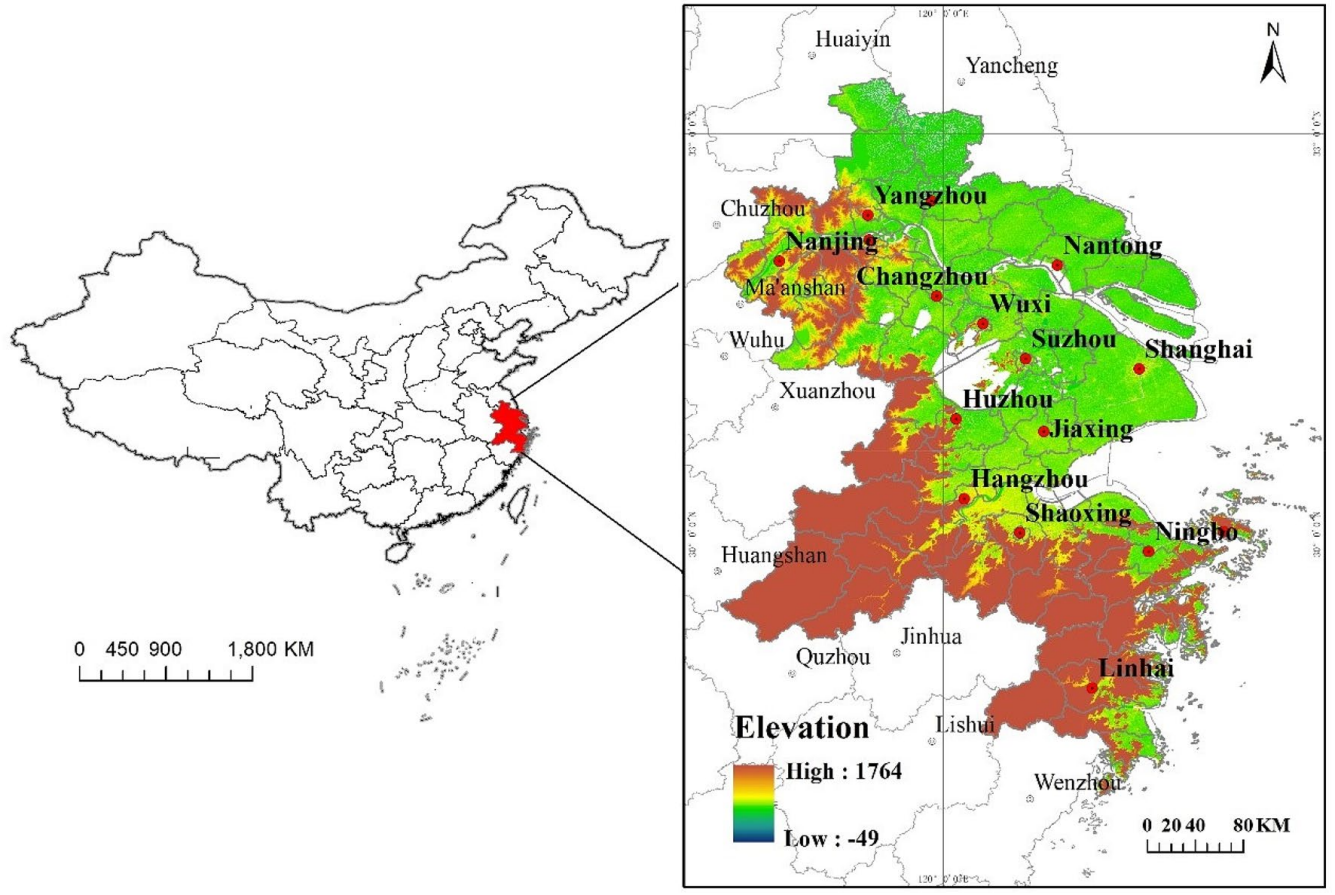
Geographic location of the YRDUA and elevation of the region which derived from Shuttle Radar Topography Mission (SRTM) image in 2017.

UHI intensity is often assessed by the urban–rural dichotomy method, that is, the difference between real-time or cumulative surface temperature between the city and the suburbs and surrounding villages^36,37^. As a new urban development form, the urban agglomeration has the characteristics of close spatial organization structure and close economic connection. The original urban–rural dichotomy method is difficult to apply to the study of heat islands in urban agglomeration where the distance between cities is shortening or even disappearing. Because urban–rural dichotomy method considers the city as one unit, which excludes the heat produced by the areas surrounding that city^38^ and hence, is unable to point out the suburban areas, least affected by the urbanization^39^. By introducing the concept of RHI, this paper constructs a method suitable for the evaluation of SRHII from the pixel view, which is not limited to the concept and boundary of a single city and village. Taking 1 × 1 km pixel as the unit,  more 2 °C^12^ than mean land surface temperatures (LST) of the surrounding background area (present in “Methods” part) with the lowest impact of urbanization was defined as the RHI. The background was selected based on elevation, land cover, NL, and annual maximum normalized difference vegetable index (NDVI). YRDUA that one of the fastest-growing and urbanizing regions in China (Fig. 1) was selected as a case study, with a total land area of about 110,000 km^2^. Trends of the contributions of influence factors to RHI have been fully revealed from the seasonal variations, inter-annual variations, daytime-nighttime variations, and different levels of SRHII. The main purpose of this paper is to (1) perform a quantifying analysis of the spatial and temporal variability of the RHI in the YRDUA; (2) compare the seasonal, inter-annual, and daytime-nighttime differences of the relationship between the RHI intensity of different graded surfaces (2–4 °C, 4–6 °C, > 6 °C) and their influencing factors; and (3) clarify the changes in the dominant influencing factors by analyzing the relative importance of the factors that influence the RHI in 2003, 2010, and 2017. The study will provide a theoretical basis for regional thermal environment planning and sustainable development.

## Results

### Spatial and temporal variations of the SRHII at daytime and nighttime

Significant seasonal differences are observed in the SRHII in the YRDUA (Figure A1 and A2, Appendix A). In the daytime, RHI was concentrated in the Nanjing, "Su-Xi-Chang", Ningbo, Shanghai, and Hangzhou metropolitan areas. Due to the high built-up areas and PD, the distribution of surface RHI is denser and stronger than that in the north and southwest of the YRDUA. The built-up area can absorb heat and store heat energy, which makes the surface temperatures rise rapidly. In spring and autumn, the spatial distribution of the RHI in spring or autumn was similar to that in summer except the spatial extent was tapered. However, the RHI gradually shrinks and transfers to the southern area of the YRDUA in winter, such as Linhai and Ningbo City, which is due to the relatively high solar radiation of the geographic location of the southern cities. The distance of the RHI is gradually shortened between cities and even into one piece from 2003 to 2017 due to long-term urban expansion and rapid growth of construction land (Figure A1, Appendix A). In the nighttime, the spatial pattern of the RHI is very different from that of the daytime. RHI mainly concentrates on Taihu Lake, Dianshan Lake, Ge Lake in the center part, Hongzhe Lake in the northwest, and Qiandao Lake in the southwest. Because water has a high specific heat capacity, it has the function of preserving heat at nighttime. Some cities like Shanghai, Hangzhou, and Nanjing have the strongest heat island in winter and the weakest heat island in summer. Urban areas usually have dense buildings, PD, and energy emissions, so there are more energy emissions at night. High surface albedo in urban areas at night leads to lower heat storage^4,40^ and ultimately resulting in smaller UHI at nighttime (Figure A2, Appendix A).

**Figure 2:**
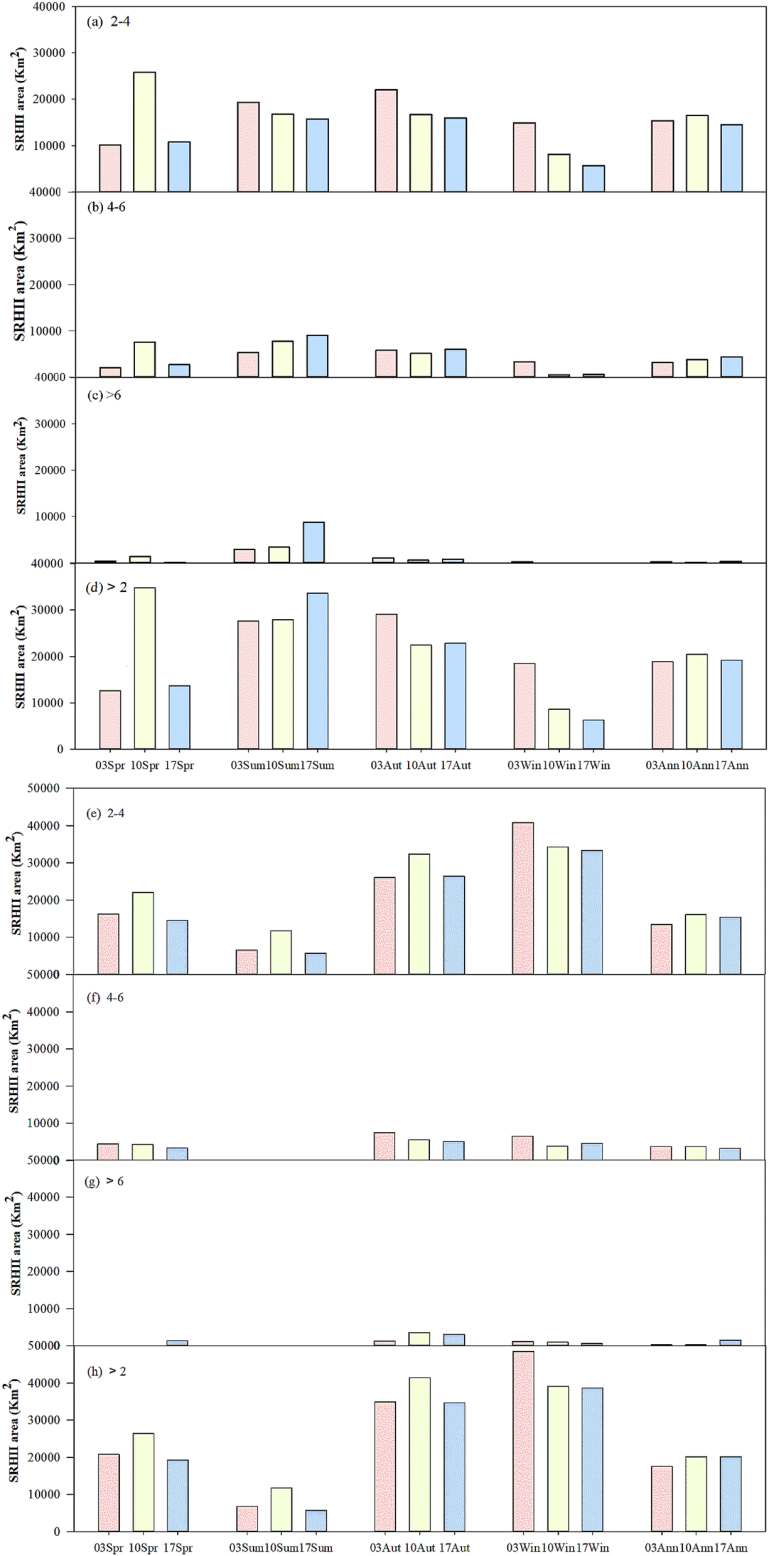
Seasonal and inter-annual variations of the SRHII during the daytime (**a**–**d**) and nighttime (**e**–**h**) of the YRDUA.

From spring to summer and then summer to winter, RHI increases first and then decreases, and it reaches a peak in summer. For example, the proportion of the RHI was 12.65%, 31.03%, 21.12%, and 5.49% in spring, summer, autumn, and winter in 2017, respectively (Fig. 2d). An upward trend in the area of the RHI is observed from 2003 to 2017 in summer. In detail, the proportion of the heat island zone is 21.74%, 22.17%, and 31.03% in the summer of 2003, 2010, and 2017, respectively (Fig. 2d). It is because the urban areas of YRDUA have increased from 3571.01 km^2^ to 8760.26 km^2^ in 2003 and 2017, respectively (Figure B1, Appendix B). Moreover, the area of the medium heat island and strong heat island increased by 41.08% and 66.40% from 2003 to 2017 (Fig. 2b,c). A gradual decreasing trend is observed for the four grades of the SRHII (2–4 °C, 4–6 °C, > 6 °C, > 2 °C) in winter from 2003 to 2017 (Fig. 2a–d). The area of the RHI in winter was 18,481 km^2^, 8640 km^2^, and 6280 km^2^ in 2003, 2010, and 2017, respectively (Fig. 2d). Vegetation coverage is low in winter and bare soil is formed after harvest. It leads to the RHI decrease in winter. The above results indicated that the SRHII became increasingly hot in summer and increasingly cold in winter and that the trend became more obvious as the SRHII increased in the ranges of 2–4 °C, 4–6 °C, > 6 °C. However, the seasonal variation of the RHI in the nighttime is opposite to that in the daytime. From spring to summer and then to winter, the area of the RHI decreases first and then increases, and it falls in the lowest value in summer (Fig. 2e–g). For example, the area of RHI is 19,209 km^2^, 5659 km^2^, 34,621 km^2^, and 38,596 km^2^ in spring, summer, autumn, and winter in 2017, respectively (Fig. 2h). The annual average of RHI regular increases, with values of 17,510 km^2^, 20,042 km^2^, and 20,097 km^2^ in 2003, 2010, and 2017, respectively (Fig. 2h).

### Relationship between the SRHII and influencing factors

**Figure 3:**
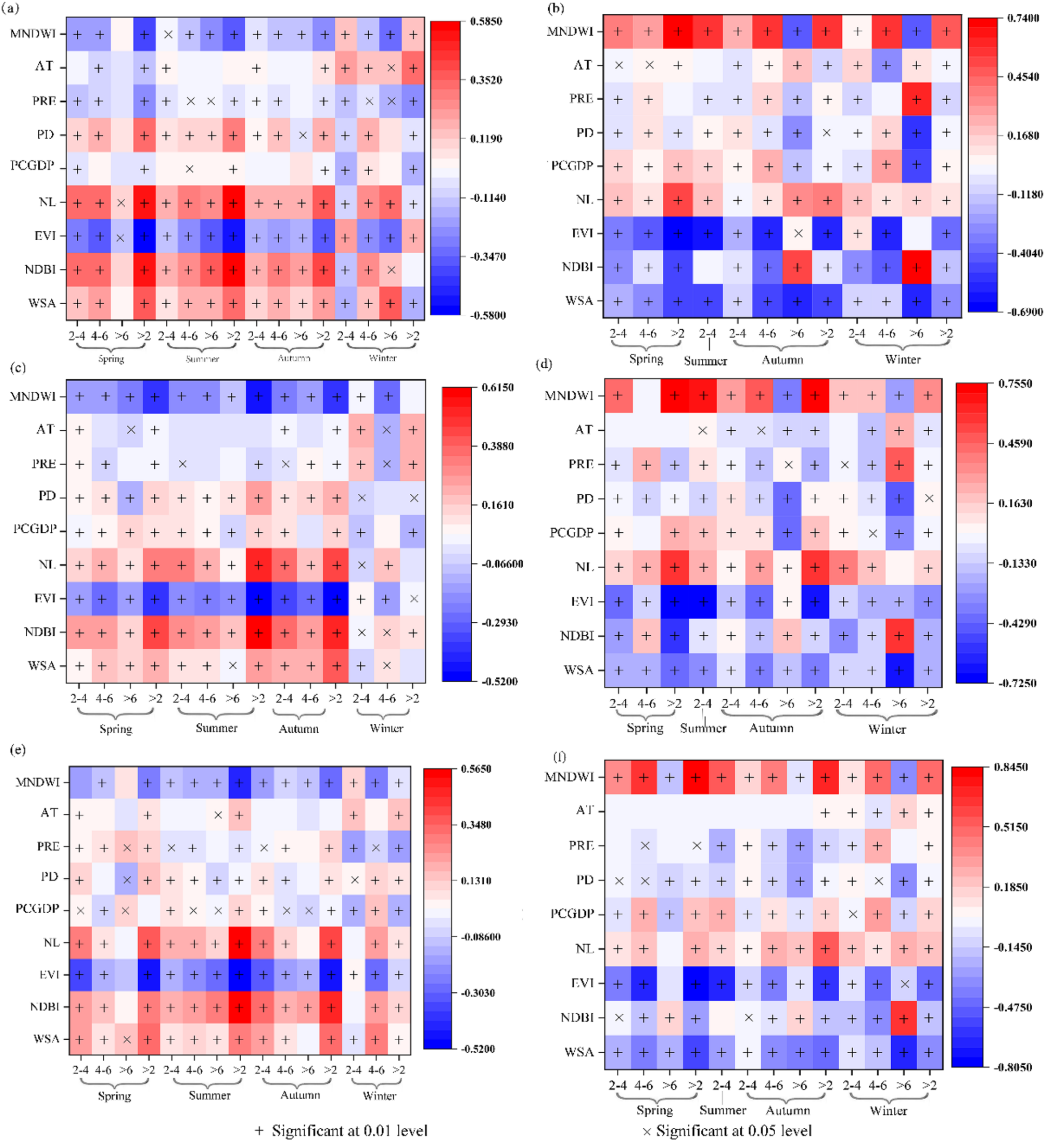
Pearson correlation coefficients of different grades of the SRHII (2–4 °C, 4–6 °C, > 6 °C, > 2 °C) during the daytime (**a**,**c**,**e**) and nighttime (**b**,**d**,**f**) of 2003, 2010 and 2017.

Results showed surface biophysical factors have a higher correlation with SRHII than socio-economic factors and climate factors in the day and night. NDBI and EVI have a stronger effect on SRHII than other biophysical factors in the day. NDBI showed a significant positive correlation with SRHII, while EVI showed a negative correlation with SRHII. In detail, NDBI (r = 0.567, p < 0.001) was the highest correlation with RHI in summer, followed by EVI (r = − 0.54, p < 0.001) (Fig. 3a). The correlation of EVI was highest in spring (r = − 0.577, p < 0.001). NDBI also showed the highest correlation in autumn (r = 0.425, p < 0.001) (Fig. 3c). White-sky Albedo (WSA) has a positive correlation in each season. MNDWI has a positive effect on the cooling heat environment in the day for its high specific heat capacity. However, the correlation of biophysical factors in the day was different at night. MNDWI has the highest correlation (positive correlation) with SRHII, followed by the EVI (negative correlation) at night. The correlation between the MNDWI and SRHII in spring, summer, autumn, and winter was 0.844, 0.558, 0.725, and 0.492 (Fig. 3f) respectively. NDBI has a low degree of interpretation of SRHII at night compared to other biophysical factors, indicating that the effect of building density on SRHII at night was weak. Zhou^11^ also showed that a weak negative correlation between UHI intensity and built-up intensity was observed in the YRDUA at night. WSA was negatively associated with SRHII at night. Compared with the conclusions in the daytime show that during the day, water bodies have a cooling effect on RHI in the day and a warming effect at night while the trend of WSA was the opposite. Vegetation has the effect of relieving the thermal environment around the clock. It indicated that water bodies were not the best choice for relieving the thermal environment. In addition, As SRHII increases from weak (2 °C < SRHII ≤ 4 °C), medium (4 °C < SRHII ≤ 6 °C) to strong (SRHII > 6 °C), the correlation coefficient (r) of WSA and NDBI increases while EVI and MNDWI have opposite trends. It indicated that controlling building density and changing the building materials, increasing the proportion of vegetation and water bodies can mitigate RHI, especially in areas with high and extremely high temperatures.

In the socio-economic factors layers, anthropogenic heat emission can’t be ignored in the spatial distribution of RHI in the day. It has the highest correlation with RHI in spring, summer, and autumn, with 0.534, 0.582, and 0.366, respectively. PD has a positive correlation to RHI with (r = 0.278, p < 0.001) (Fig. 3a). Per capita gross domestic product (PCGDP) has no significant correlation with the RHI in all seasons, indicating that the influence of economic development level on the change of RHI is weak. At night, anthropogenic heat emissions in spring and summer were also the most relevant to SRHII, at 0.28 and 0.295 (Fig. 3f), respectively. The correlation between PCGDP and SRHII at night was highest in autumn and winter, at 0.183 and 0.178, respectively. PD has a weak effect on SRHII at night. With the increase of SRHII from weak, medium to strong, the correlation increased between anthropogenic heat emission and SRHII, which means that the effect of artificial heat discharge becomes a more important role with the increase of SRHII.

In the climate factors layers, air temperature is positively correlated with SRHII while PRE is negatively correlated with SRHII during the day. For winter, these factors are closely related to SRHII distribution than biophysical and socio-economic factors. Specifically, AT (r = 0.343, p < 0.001) became the highest correlated factor with SRHII in winter, followed by PRE (r = 0.234, p < 0.001) (Fig. 3a). The correlation of AT and PRE and SRHII was weak at night in each season (Fig. 3f).

In summary, NDBI, EVI, and anthropogenic heat emission have related closely to RHI and present seasonal variations in the day, with summer > spring > autumn. Climate (AT and PRE) factors can explain more spatial differences of RHI in winter. Water bodies and surface albedo have a negative and positive effect on the SRHII in the daytime and nighttime. With the increase of SRHII from weak, medium to strong, the correlation coefficient (r) of SRHII and albedo, NDBI, and anthropogenic heat emission increases while EVI and MNDWI have the opposite trends.

### Changes in dominant factors of the SRHII

**Figure 4:**
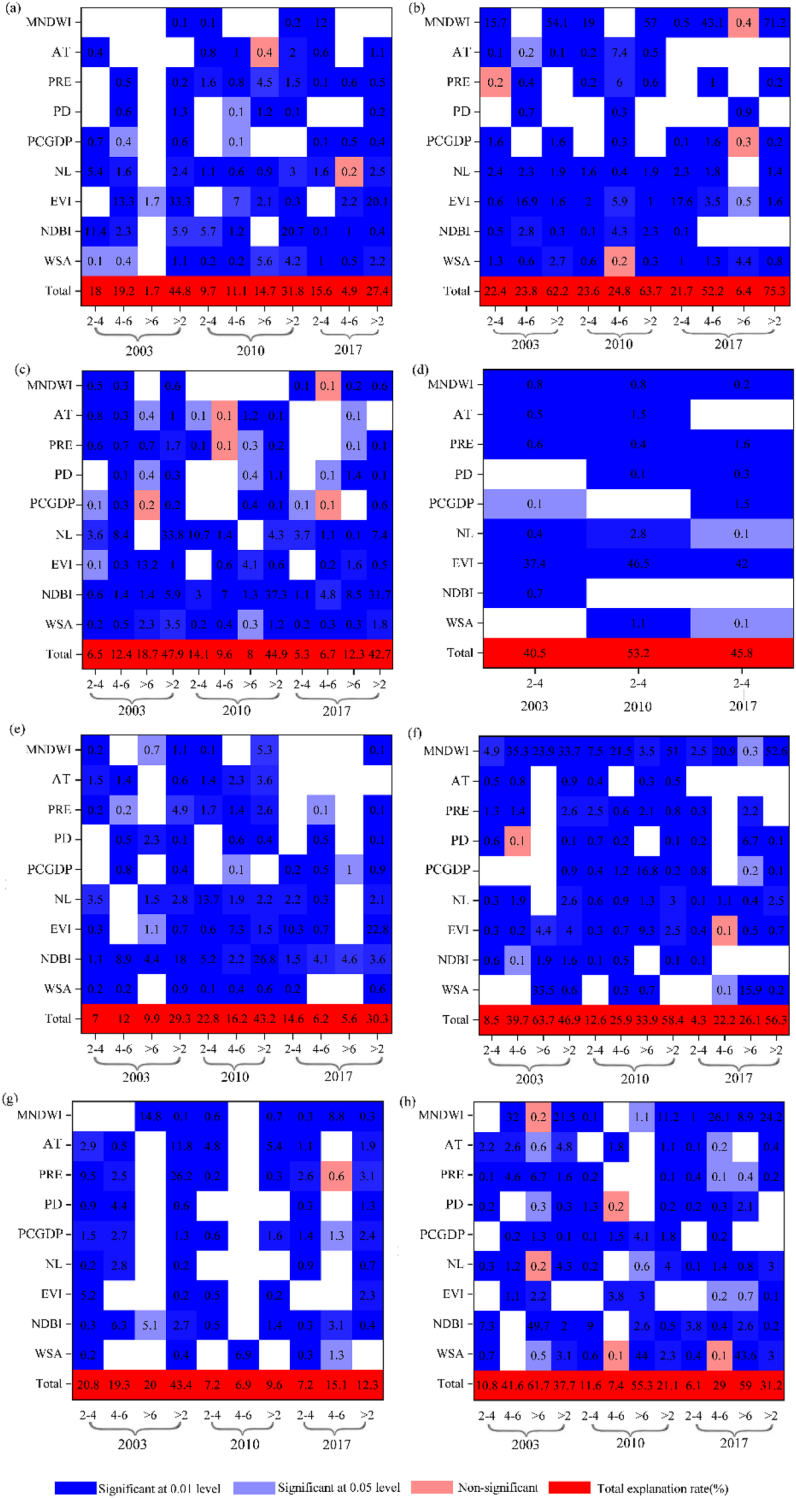
Explanation degree of the stepwise linear regression analysis of the SRHII (°C) and impact factors during the daytime (**a**,**c**,**e**,**g**) and nighttime (**b**,**d**,**f**,**h**) in spring (**a**,**b**), summer (**c**,**d**), autumn (**e**,**f**), and winter (**g**,**h**).

The results of the stepwise regression show that the EVI and NDBI have the largest independent contribution relative to the other factors in the variation of SRHII except in winter. The most important independent variable factor affecting the SRHII is the EVI in the spring daytime of 2003, and it had an explanatory rate of 33.3% (Fig. 4a). However, the dominant explanatory factor changed to the NDBI (explanatory rate is 20.7) in the spring daytime of 2010 and the EVI (explanatory rate is 20.1%) in the spring daytime of 2017. The total explanation rates increased as the SRHII increased. For example, the total explanatory rates affecting the SRHII in 2010 spring daytime are 9.7%, 11.1%, and 14.7% in the range of 2–4 °C, 4–6 °C and > 6 °C, respectively. NL has the largest interpreted independent contribution (33.8%) on the summer daytime of 2003, although the dominant influencing factor shifted to the NDBI (37.3% and 31.7%) in 2010 and 2017. In the summer daytime of 2017, the total explanatory rates were 5.3%, 6.7%, and 12.3% for SRHII in the range of 2–4 °C, 4–6 °C and > 6 °C, respectively (Fig. 4c).

For autumn daytime, the most important independent factor affecting the SRHII is the NDBI, whose explanatory rate was 18% in 2003. The dominant explanatory variable was also the NDBI in 2010, and its explanatory rate was 26.8%. However, the largest explanatory factor shifted to the EVI (explanatory rate was 22.8%) in 2017 (Fig. 4e). In winter, the total explanatory rate fluctuated in 2003, 2010, and 2017. Compared with other seasons in the daytime, PRE had a large explanatory rate (26.2%) for the SRHII in the winter of 2003. In 2010, the main explanatory variable was AT (5.4%) (Fig. 4g).

At night, MNDWI was the dominant explanatory variable in spring, autumn and winter, while EVI was the main explanatory variable in summer. In detail, MNDWI showed the largest interpreted independent contribution to the SRHII in spring, and its explanatory rate was 54.1%, 57%, and 71.2% in 2003, 2010, and 2017, respectively (Fig. 4b). In the summer, the dominant explanatory parameter was the EVI in 2003, 2010, and 2017, and it explained 37.4%, 46.5%, and 42%, respectively (Fig. 4d). MNDWI also had the largest interpreted independent contribution in 2003, 2010, and 2017, and it explained 33.7%, 51%, and 52.6%, respectively in autumn (Fig. 4f). In winter, the MNDWI had the largest interpreted independent contribution in 2003, 2010, and 2017, and its explanatory rate was 21.5%, 11.2%, and 24.2%, respectively (Fig. 4h). The total explanatory rates also increased as the SRHII increased from weak, medium to strong in each season.

In summary, NDBI, EVI, and anthropogenic heat emission were the key parameters for determining the daytime RHI when the relative temperature was high in YRDUA, while AT and PRE were the dominant explanatory variable in winter. The total explanatory rates affecting the daytime SRHII present seasonal variations, with summer > spring > autumn > winter. The dominant influencing factor was the MNDWI in spring, autumn and winter, while EVI had the largest contribution in summer at night.

## Discussion

The spatial and temporal variations of the RHI in the YRDUA show that the heat island zone has gradually expanded and even become agglomerated (Figure A1 and A2, Appendix A). UHI is no longer a single local phenomenon and has become an aggregated RHI, and it will cause a thermal environment agglomeration effect. Therefore, it is necessary to protect natural forests and build green infrastructure to reduce the RHI effect. Surface biophysical, socio-economic and climate factors have certain effects on this. The Pearson's correlation coefficients in this study showed that the NDBI (r = 0.563 p < 0.001), NL (r = 0.536, p < 0.001), and EVI (r = − 0.516, p < 0.001) have a high correlation with the change of the SRHII in summer, spring and autumn (Fig. 3e). These results are consistent with those of previous studies^19,41^, the correlation coefficients of the NDBI, EVI, and NL remained approximately 0.5. Climate (AT and PRE) has a high correlation with SRHII, which indicated the spatial distribution of RHI was more controlled by biophysical factors with the higher the temperature. When the temperature of the study area decreases (such as winter), it is more vulnerable to climatic factors. Moreover, as the SRHII increased from weak, medium to strong (Fig. 3a,c,e), and the warming effect of built-up density, albedo, and anthropogenic heat emissions becoming more significant, and the cooling effect of vegetable activity and water bodies becoming more important. It is consistent with the Jia and Zhao’s^42^ studies. The study pointed that as the intensity of urban development increases from low to medium to high, urban greenspace represents an increasingly important factor for alleviating LST. In addition, the MNDWI, EVI, and WSA had a strong impact on the SRHII at nighttime (Fig. 3b,d,f). MNDWI harms SRHII in the day while having a positive effect at night, which indicated that water bodies absorb heat during the daytime and release heat at nighttime.

**Figure 5:**
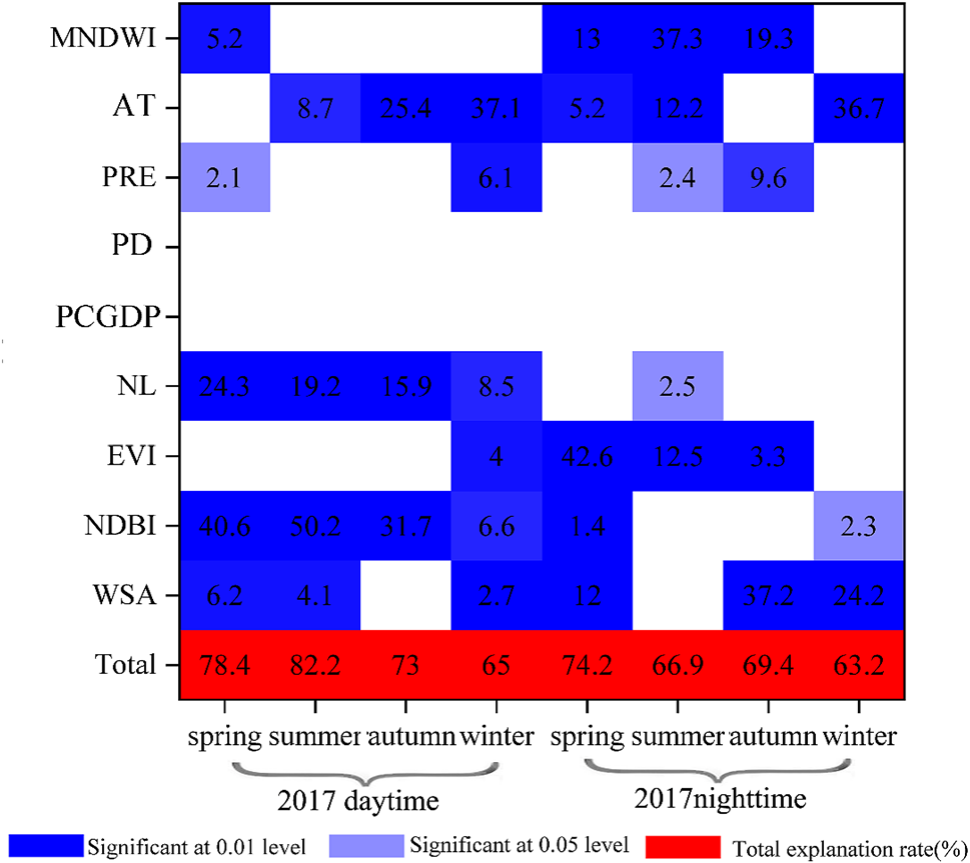
Explanation rate of influencing factors for the SRHII (°C) at the urban scale.

The stepwise regression model showed that the NDBI (31.7% for contributing rate) and EVI (22.8%) were the dominant influencing factors for the RHI in the daytime (Fig. 4c,e). The explanatory rate of the NDBI and EVI was slightly lower than that of previous studies^19,29^. To illustrate whether the difference of explanatory rates is due to the selected influencing factor, we used the same influencing factors and the same method to calculate it again except the pixel scale was changed to the city scale. The results showed that the explanatory rate of the NDBI reached 50.2% and the total explanatory rate reached 82.2% in the daytime in the summer of 2017 (Fig. 5). This finding indicates that the scale and extent have a certain effect on the factors that influence the RHI. This result is similar to that of Luan et al.^43^. Therefore, it is necessary to control the expansion of construction land as much as possible, original sand and gravel surfaces should not be covered and the area of the impervious surface should be controlled. Permeable trail or blue-green infrastructure should replace impervious surfaces. Changing building materials is another way to control the RHI intensity. New environmentally friendly building materials (e.g., fiber-reinforced plastics and bamboo-glass fiber composite building materials) can replace traditional building materials, which are mainly composed of steel, glass, and concrete. Moreover, MNDWI plays a key effect on the RHI at night, which is similar to previous conclusions^21,44^. Water bodies have a negative effect on SRHII in the day and a positive effect at night. The findings indicated that water bodies are a cooling source in the daytime but also a heat source at nighttime. Therefore, it is necessary to consider the heat island effect of water bodies at nighttime in urban or regional thermal environment planning. Hence, this study recommends that green vegetation is more suitable for cooling the thermal environment.

Certain limitations were observed in this paper. First, the influence of data quality and some specific meteorological conditions may lead to certain uncertainties. Thus, the image quality was tested, and the average temperature was used. Second, there are multi-collinearity problems among the influencing factors. Multiple statistic technique was used to improve the accuracy in the Pearson correlation coefficients analysis. The stepwise regression is used in this paper, which eliminates multiple collinearities and selects optimal dominant factors. The coefficient of variance expansion VIF test the multiple collinearities between variables was used. When VIF > 10, it means that there is a multiple collinearity problem between variables, and the redundant variables are eliminated. Third, the extremely high-temperature zone has certain pixels that disappear in a specific season, which leads to low explanatory rates and sometimes non-significant correlations. Moreover, the spatial resolution has a certain effect on the study of the UHI^45,46^. The spatial resolution of the data used in this paper is 1 km. A certain deviation in the results may have occurred because of differences in the coarse spatial resolution. Hence, spatial and temporal fusion methods need to be used to obtain more detailed results. Even the results based on long-term changes in SRHII are accepted in the field and show the trends of the contributions of biophysical and socioeconomic elements to RHI. The spatial heterogeneity and collective effect of SRHII among cities are also necessary to solve in further study.

## Conclusions

In this study, the SRHII was calculated as the difference between each pixel and background based on the elevation, land cover, annual maximum NDVI, and NL. The trends of the contributions of influencing factors to the SRHII were studied using Pearson's correlation coefficients and a stepwise regression model. The spatial and temporal variation of the RHI indicated that the SRHII strengthened in summer and became colder in winter and the heat island zone became more concentrated/agglomerated and started to become one unit. The NDBI, NL, and EVI have a high correlation with the SRHII and present seasonal variations, with summer > autumn > spring. When the SRHII increased (the range of 2–4 °C, 4–6 °C and > 6 °C), the Pearson’s correlation coefficients of the NDBI, WSA, NL increased and the EVI and MNDWI decreased in the daytime. Moreover, the MNDWI, EVI, and WSA had an important influence on the SRHII at nighttime. The NDBI and EVI were the dominant factors for the surface RHI during the daytime. This indicates that controlling the building density and vegetation can effectively adjust the RHI environment. The MNDWI has a dominant influence on the SRHII in the nighttime, which means that the role of water bodies on cooling temperatures in urban or regional areas is relatively limited. Based on the above conclusions, we recommend that the built intensity and vegetation should be the focus of planning. It is necessary to change the building materials and build green roofs (and green space) to improve the regional thermal environment. This study can provide theoretical bases for regional environment improvement and sustainable development.

## Methods

### Data sources

**Figure 6:**
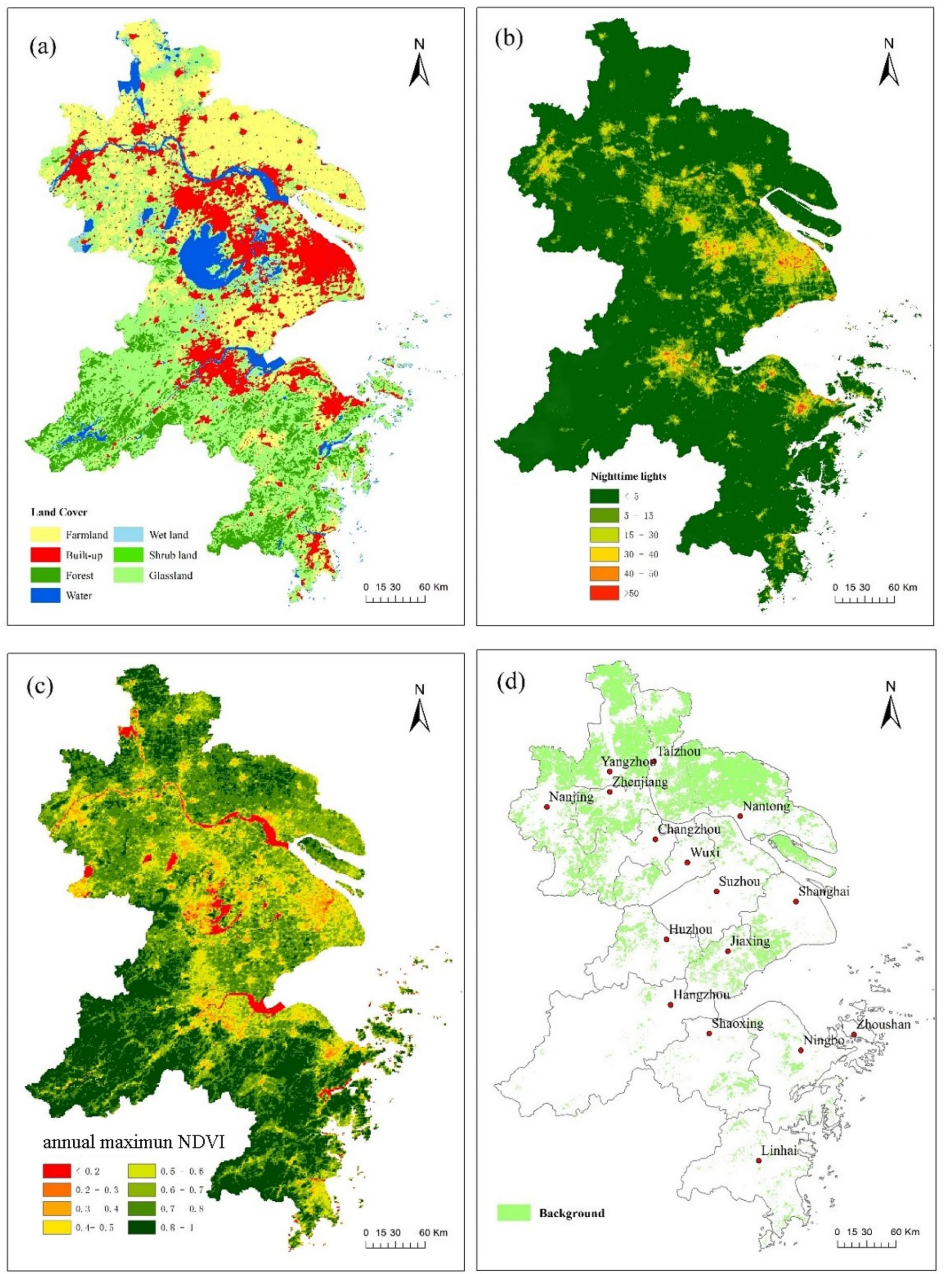
(**a**) Land-cover map derived from MODIS Land cover type (MCD12Q1) images. (**b**) NL map derived from the Visible Infrared Imaging Radiometer Suite (VIIRS). (**c**) Annual maximum NDVI derived from MODIS Normalized Vegetation Index Product (MYD13A3) image. (**d**) Background in YRDUA were extracted by elevation, land cover, annual maximum NDVI and NL.

LST data from 2003, 2010, and 2017 were derived from the MODIS/Aqua land surface temperature/emissivity 8-day L3 Global 1 km SIN Grid V006 (MYD11A2) and 276 MODIS images with cloud covered less than 50% were downloaded from the official NASA website (https://earthdata.nasa.gov/) with a 1 km spatial resolution. DEM data were derived from Shuttle Radar Topography Mission (SRTM) data (http://srtm.csi.cgiar.org/SELECTION/listImages.asp), with a spatial resolution of 1 km. NDVI data were obtained from the MODIS Normalized Vegetation Index Product (MYD13A3). NDBI and MNDWI data were obtained from the MODIS surface reflectance Product (MYD09A1) with a 1 km spatial resolution. NL data were obtained from the Operational Line scan System (OLS) (https://www.ngdc.noaa.gov/eog/dmsp/downloadV4composites.html) and the Visible Infrared Imaging Radiometer Suite VIIRS (version 1) (https://www.ngdc.noaa.gov/eog/viirs/download_dnb_composites.html). EVI data were obtained from the MYD13A2 products. Albedo data were from the MODIS Bidirectional Reflectance Distribution Function (BRDF)/Albedo Parameter products (MCD43A3) because the WSA had the same relationship with the black sky albedo (BSA). Therefore, the WSA over shortwave broadband with 1 km spatial resolution was chosen. PD and PCGDP in districts and counties were derived from the *Shanghai Statistical Yearbook*, *Statistical Yearbook of Jiangsu Province,* and *Statistical Yearbook of Zhejiang Province*^47^. Then, geospatial information visualizations technology^48^ was used to generate a grid layer at 1 km resolution. AT and the PRE data for 2003, 2010, and 2017 were released by the China meteorological data service center (http://data.cma.cn/en) of Shanghai, Jiangsu, and Zhejiang Province. After obtaining the station data for AT and PRE, a spatial interpolation approach (ANUSPLIN)^49^ with the DEM data at 1 km resolution as a covariate variable was used to generate continuous surface for seasonal AT and PRE. Land cover data were obtained from MODIS/Terra Aqua Land cover type (MCD12Q1) with 500 m spatial resolution in the YRDUA. The global vegetation classification scheme of the International Geosphere-Biosphere Program (IGBP) was used, including the following seven land cover types: built-up, forest, scrublands, grassland, wetland, farmland, and water (Fig. 6a). To verify the accuracy of the land cover map, we selected 200 random points and compared them with the actual features in Google Earth at the same time to judge its accuracy. The inspection precision is 0.87, 0.81 and 0.83 in 2003, 2010 and 2017, respectively. The inspection precision is large than 0.8, which satisfied the requirements of the analysis^19,50^. MATLAB and ArcMap10.7 software (https://www.arcgis.com) was used to process the above data.

In addition, March, April, May were defined as spring, June, July, and August as summer, September, October, and November were defined as autumn, and December, January, and February are winter.

### Methods for calculating the SRHII

Beyond the boundaries of a single city, SRHII was calculated as the actual surface temperature difference of each pixel (1 km × 1 km) relative to that of the background with the lowest effect of urbanization. The background was selected based on elevation, land cover, annual maximum NDVI, and NL.

Firstly, elevation difference below 50 m relative to urban areas was selected while determining the background (Fig. 1). There are some articles^51,52^ which found that LST and elevation have a positive linear trend, most of the areas in YRDUA are about 10 m above sea level, with low hills scattered at an altitude of 200 m. Therefore, the threshold of 50 m can reduce the deviation of heat island caused by elevation as much as possible. Secondly, the farmland has been selected as one of the conditions for the background (Fig. 6a), since farmland is the natural environment near the urban area^18,20^. LST for different types of land cover in the daytime and nighttime was shown in Table B1 and Table B2, Appendix B*.*

Third, the value of the NL less than or equal to 15 was selected as the background, because these regions were not affected by human activity^53,54^.NL has been widely used in the research of urbanization. Figure 6b shows the spatial distribution of the NL's value of the YRDUA. Lastly, the area where the annual maximum NDVI (Fig. 6c) is greater than or equal to 0.7 was selected. The purpose to choose the annual maximum NDVI is to exclude the low vegetation cover area including the water body in the YRDUA. The above four conditions are satisfied simultaneously, and the background values of the YRDUA with 1 km resolution were extracted (Fig. 6d).

SRHII based on the background was proposed. The formula is as follows:

1 $${SRHII}_{i}={\text{T}}_{i}-\frac{1}{n}\sum _{1}^{n}{T}_{B}$$

where SRHII_*i*_ is the surface RHI intensity of the *i* pixel in the image (°C), ${T}_{i}$ is the land surface temperature (LST) (°C) of the *i* pixel, n is the number of the backgrounds in the study area, and ${T}_{B}$ is LST for background pixels (°C).

**Figure 7:**
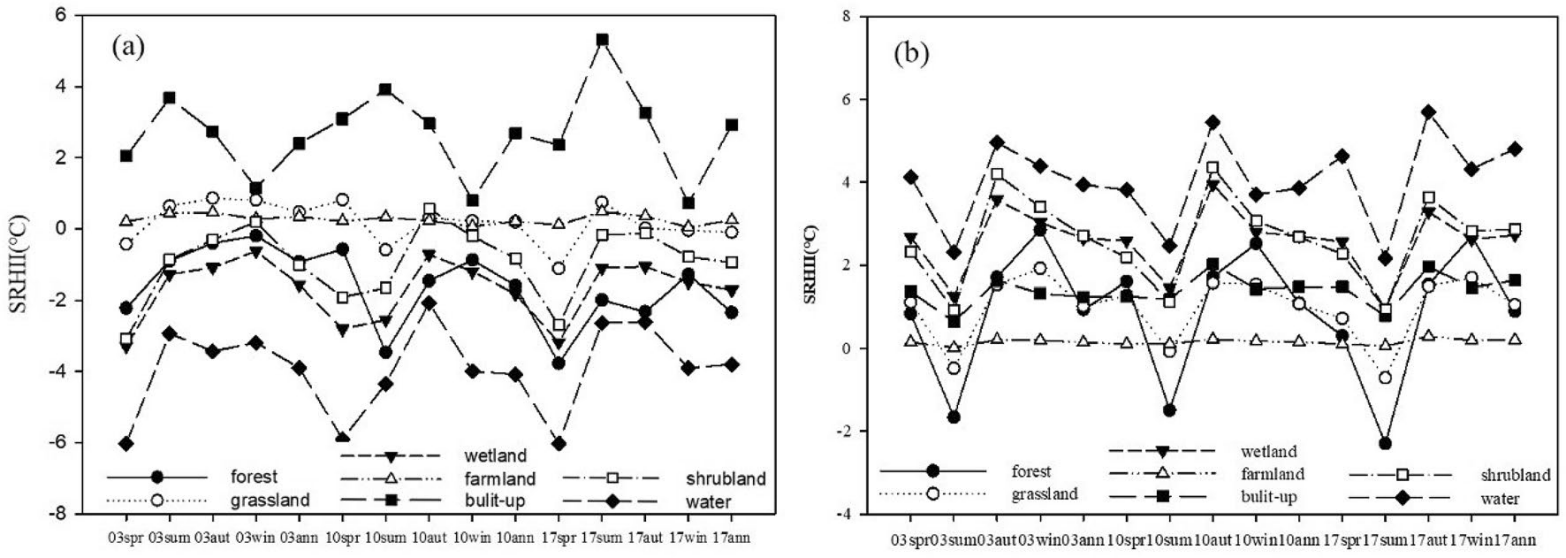
(**a**) Daytime and (**b**) nighttime SRHII (°C) for different land-cover types in the YRDUA.

To divide the SRHII, we extracted the SRHII for different land cover types. The SRHII of the built-up land is more than 2 °C in the day and night. The SRHII on the day of built-up land reached the highest value of 5.3 °C in summer 2017 (Fig. 7). The SRHII of grassland and farmland in the day is approximately 0 °C, and it shows relative stability in each season. The SRHII of shrubland and wetland was − 4 °C and 0 °C, respectively. The cooling effect of water bodies has the lowest SRHII with − 6 °C. According to the SRHII of different land types and the results of previous research^12,39^, SRHII of more than 2 °C were defined as RHI. To further reveal the changes of the SRHII, the SRHII is further divided into weak heat island (2 °C  < SRHII ≤ 4 °C), medium heat island (4 °C < SRHII ≤ 6 °C) to strong heat island (SRHII > 6 °C).

### Influencing factor selection

Surface biophysical factors have significant effects on RHI. The surface heat storage is related to the absorption (reflectivity) of solar energy during the daytime and the thermal properties (thermal capacity and thermal conductivity) of urban surfaces (building density, height, material, and pavement material). WSA was positively correlated with the UHI intensity during the daytime^16,17^. EVI is widely used to characterize vegetation cover. The distribution between latent heat flux and sensible heat flux is regulated by vegetation coverage and per unit vegetation permeability, and there is a close correlation between plant transpiration and urban surface temperature^12^. MNDWI was proposed by Xu^55^ was used to characterize water bodies. MNDWI has a certain effect on the UHI because of its large specific heat capacity. NDBI was selected as an index to characterize urban architecture, and it has a significant relationship with RHI^56^. Thus, WSA, EVI, MNDWI, and NDBI have been selected as the surface biophysical factors affecting the RHI.

In addition, socio-economic activities exert some pressure on the urban thermal environment^43^. PCGDP and PD are features of human activity and highly affect the UHI intensity^57,58^. The anthropogenic heat emission is an important factor for the urban thermal environment, and NL was used to characterize anthropogenic heat emissions^59^. In addition, background climate is an important factor that can influence the UHI effect^60^; hence, AT and PRE were selected in this study.

### Framework and analysis process

To explore the trends of the contributions of influence factors to RHI, we investigated the relationship between the SRHII and influencing factors based on the following four dimensions: daytime-nighttime differences, seasonal differences, inter-annual differences, and different grades of SRHII. A remote sensing image of the SRHII was analyzed using the above methods. All of the influencing factors were transformed to raster data. A 1 km × 1 km grid was created to extract pixels with the SRHII and the corresponding influencing factors in ArcGIS 10.7.

**Figure 8:**
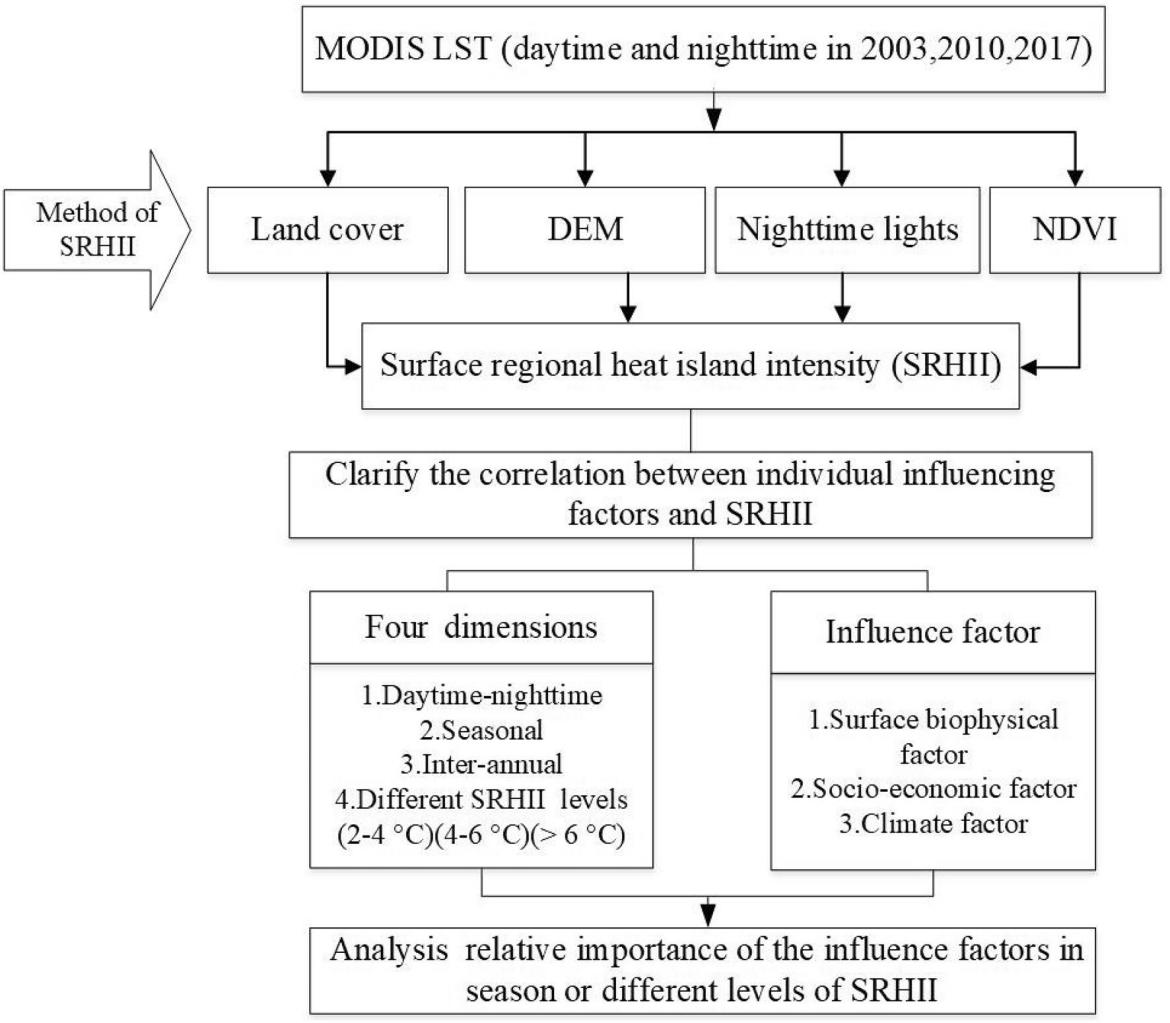
Framework of the study.

Afterward, we took the SRHII in each pixel as the dependent variables and the influencing factors corresponding to each pixel as independent variable. Then, the SRHII was divided into three grade (2–4 °C, 4–6 °C, > 6 °C) and Pearson's correlation coefficient (r) was calculated between the SRHII and different influencing factors in each season in 2003, 2010 and 2017. In addition, to determine the relative importance of the influencing factors in different seasons and different years, a stepwise regression model was used to explain the relative importance of different influencing factors. The framework of this study is shown in Fig. 8.

## Supplementary Information

Supplementary Information 1.

Supplementary Information 2.
