# Supplementary material for: Trends of the contributions of biophysical (climate) and socioeconomic elements to regional heat islands
Source: Sci Rep. 2021 Jun 16;11:12696. doi: 10.1038/s41598-021-92271-3 (PMC8209191; doi:10.1038/s41598-021-92271-3)
Supplement: Supplementary file 1 — Supplementary Information 1. [file 41598_2021_92271_MOESM1_ESM.doc]

# **Trends of the contributions of biophysical (climate) and socioeconomic elements to regional heat islands**

Shengzi Chen ^1, a^, Zhaowu Yu 2, [[1]](#footnote-2)a, *, Min Liu ^1,*^**,** Liangjun Da ^1^, Muhammad Faiz ul Hassan^3^

1 Shanghai Key Lab for Urban Ecological Processes and Eco-Restoration, School of Ecological and Environmental Sciences, East China normal university, Shanghai 200241, China

2 Department of Environmental Science and Engineering, Fudan University, Shanghai 200438, China

3. School of Electronic Sciene and Engineering, University of Electronic Sciene and technology, Chengdu 610000, China

*Correspondence: zhaowu_yu@fudan.edu.cn (Z.Yu). [mliu@re.ecnu.edu.cn](mailto:mliu@re.ecnu.edu.cn) (M. Liu)

^a^ These authors contributed equally to this work.

Appendix A: Spatial distribution of SRHII (℃) in daytime (Figure A1) and nighttime (Figure A2) in 2003, 2010 and 2017 derived from MODIS/Aqua land surface temperature image.

Figure A1. Spatial distribution of SRHII (℃) in daytime in 2003, 2010 and 2017 derived from MODIS/Aqua land surface temperature image. (MYD11A2).


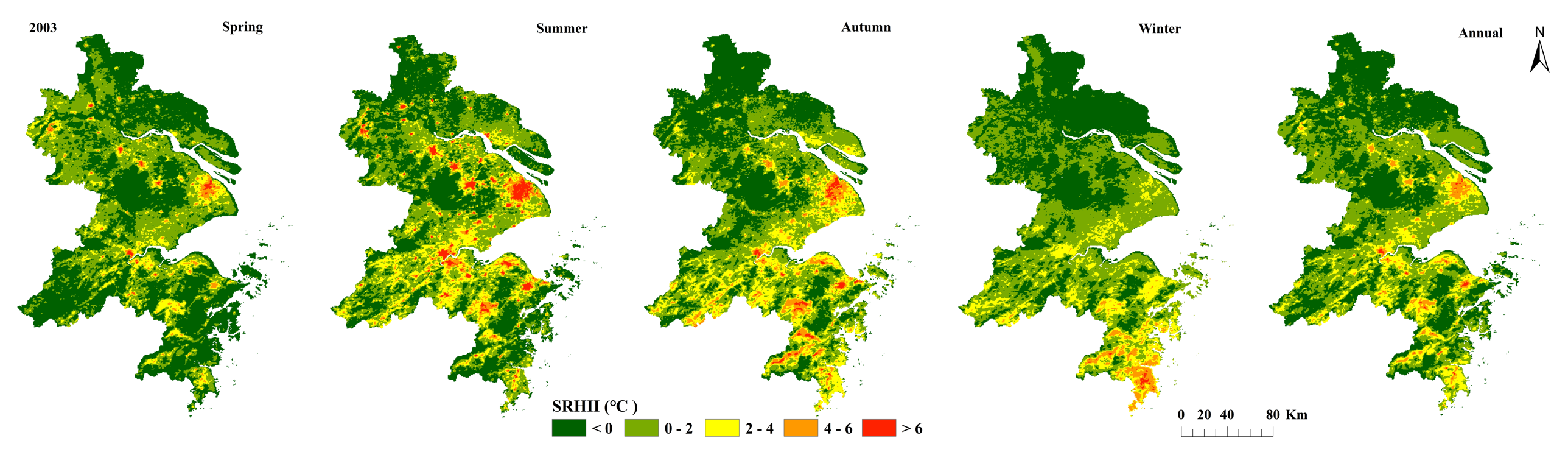


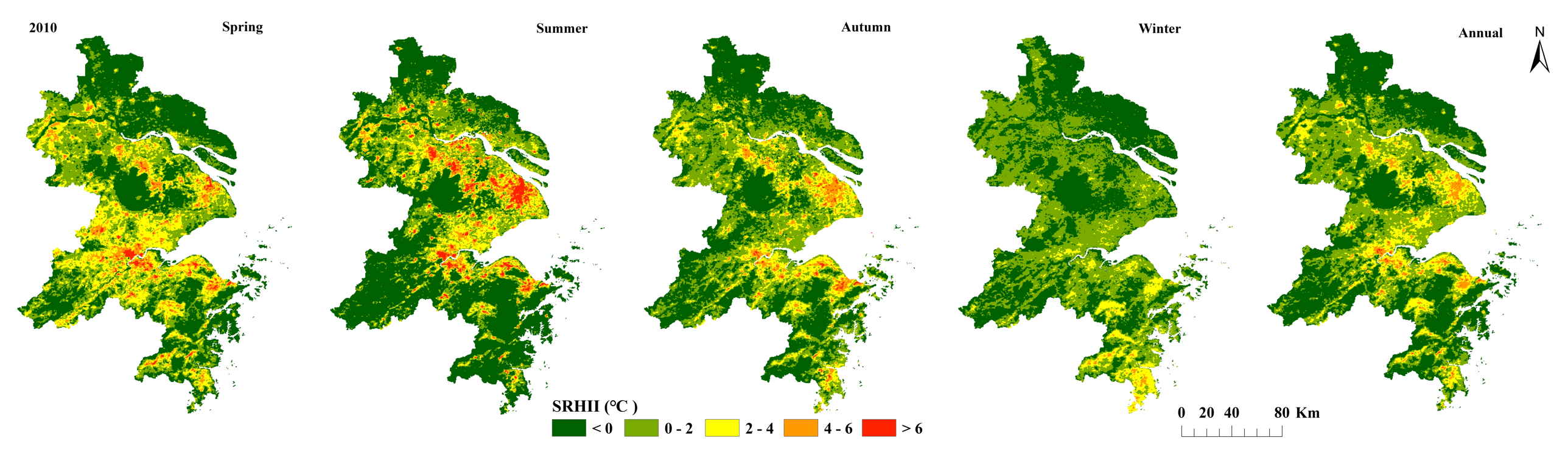


**
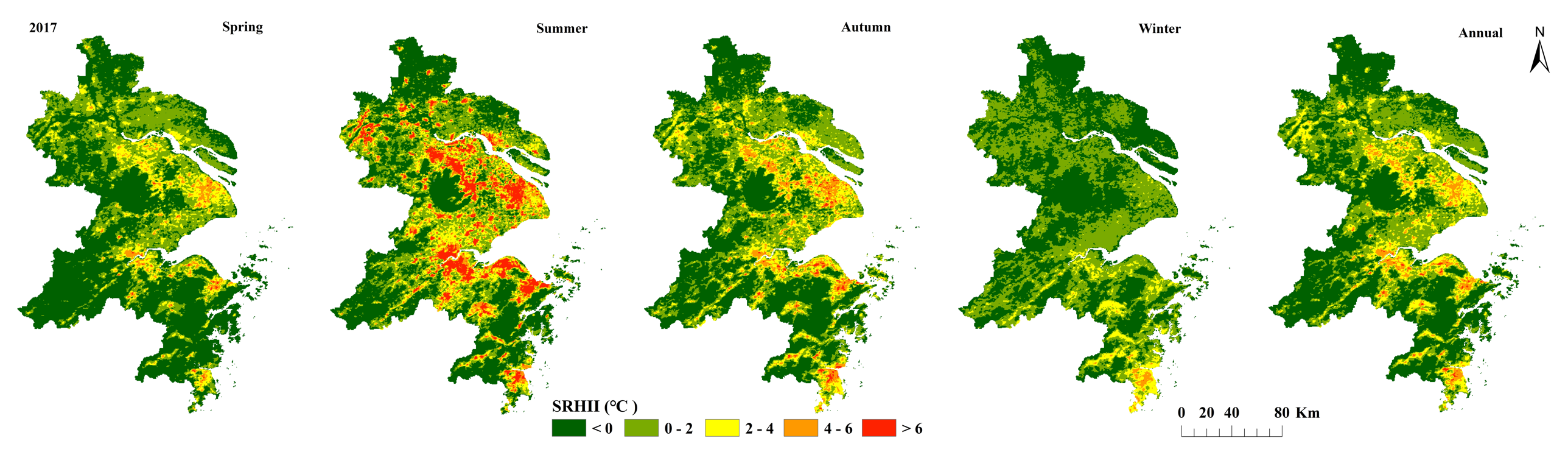
**Figure A2. Spatial distribution of SRHII(℃) in nighttime in 2003, 2010, 2017 derived from MODIS/Aqua land surface temperature image. (MYD11A2)


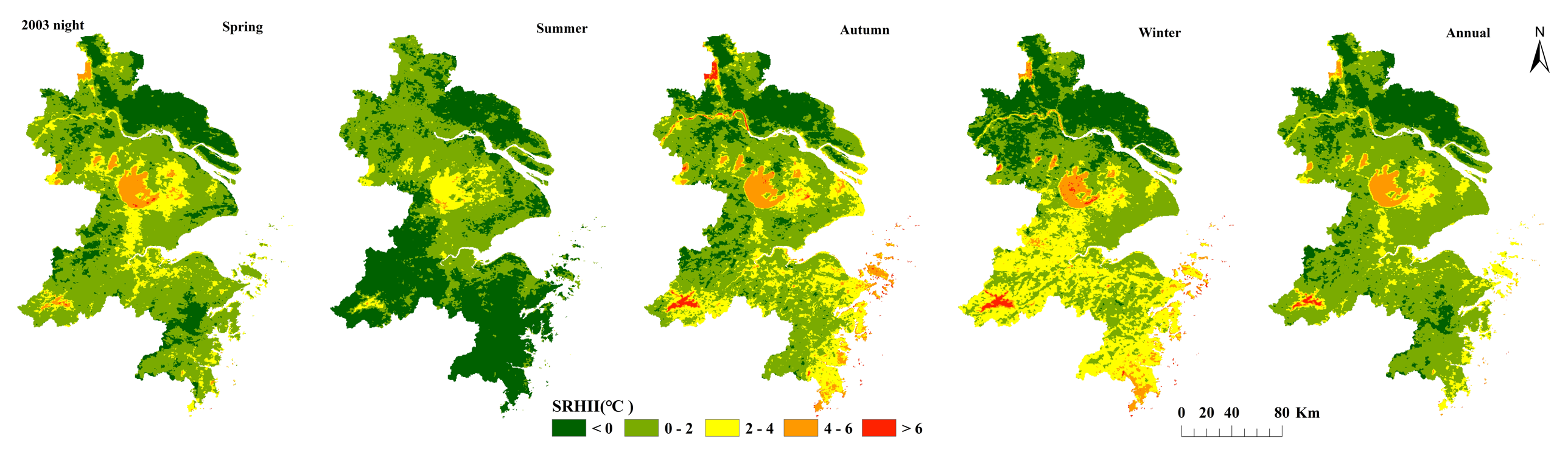


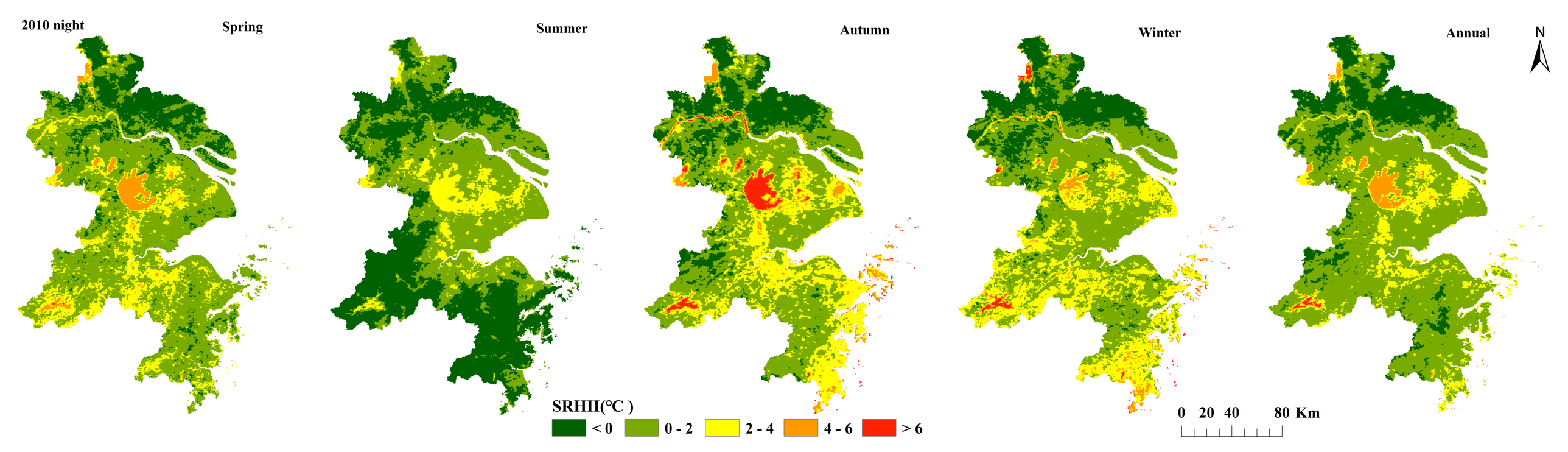


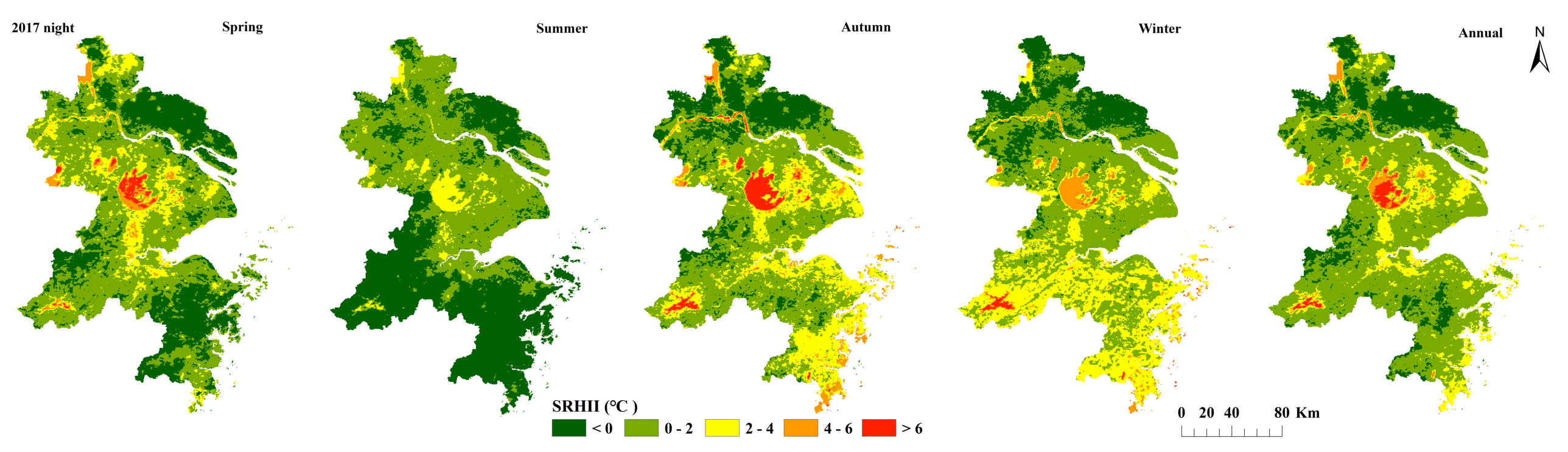


1. ^a^ [↑](#footnote-ref-2)
