# Supplementary material for: Trends of the contributions of biophysical (climate) and socioeconomic elements to regional heat islands
Source: Sci Rep. 2021 Jun 16;11:12696. doi: 10.1038/s41598-021-92271-3 (PMC8209191; doi:10.1038/s41598-021-92271-3)
Supplement: Supplementary file 2 — Supplementary Information 2. [file 41598_2021_92271_MOESM2_ESM.doc]

# **Trends of the contributions of biophysical (climate) and socioeconomic elements to regional heat islands**

Shengzi Chen ^1, a^, Zhaowu Yu 2, [[1]](#footnote-2)a, *, Min Liu ^1,*^**,** Liangjun Da ^1^, Muhammad Faiz ul Hassan^3^

1 Shanghai Key Lab for Urban Ecological Processes and Eco-Restoration, School of Ecological and Environmental Sciences, East China normal university, Shanghai 200241, China

2 Department of Environmental Science and Engineering, Fudan University, Shanghai 200438, China

3. School of Electronic Sciene and Engineering, University of Electronic Sciene and technology, Chengdu 610000, China

*Correspondence: zhaowu_yu@fudan.edu.cn (Z.Yu). [mliu@re.ecnu.edu.cn](mailto:mliu@re.ecnu.edu.cn) (M. Liu)

^a^ These authors contributed equally to this work.

Appendix B: Figure of urban expansion from 2003 to 2017 and tables of land surface temperatures for different types of land cover in the daytime (Table B1) and nighttime (Table B2), land cover data were obtained from MODIS/Terra Aqua Land cover type (MCD12Q1).

Figure B1. The bar-graph plots of urban expansion from 2003 to 2017 in the YRDUA.


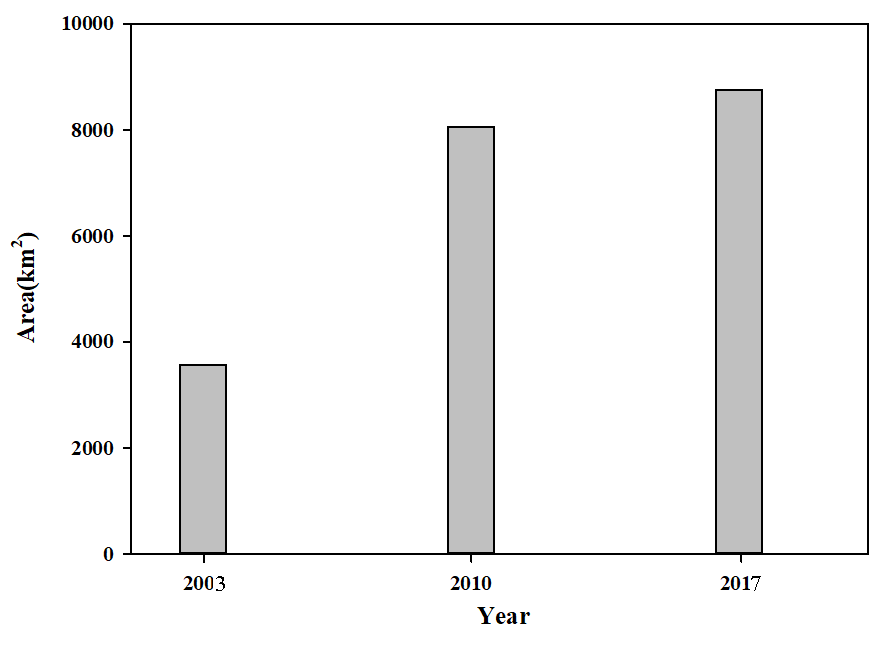


Table B1. Land surface temperatures for different types of land cover in the daytime. (Mean ± Std, ℃)

|  | Forest | Shrub land | Grassland | Wetland | Farmland | Bulit -up | Water |
| --- | --- | --- | --- | --- | --- | --- | --- |
| Jan | 12.22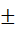1.81 | 12.02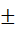2.17 | 13.22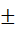2.04 | 11.21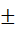1.95 | 12.36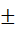2.23 | 13.95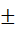2.01 | 8.47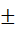2.63 |
| Feb | 14.27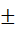1.66 | 13.83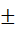2.47 | 15.74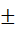1.75 | 13.5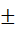1.93 | 15.74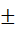1.3 | 16.55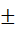1.69 | 9.98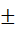2.92 |
| Mar | 15.96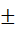1.59 | 17.42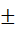2.08 | 18.56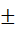1.9 | 17.04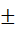1.98 | 20.09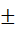1.37 | 21.33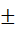1.48 | 14.12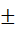2.42 |
| Apr | 24.52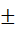1.84 | 24.6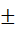2.51 | 26.83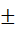2.16 | 23.98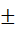2.47 | 27.12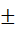1.79 | 30.51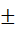2.11 | 21.28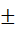2.6 |
| May | 27.19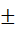1.88 | 28.81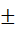2.74 | 30.37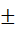2.4 | 28.38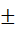2.57 | 32.19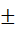1.49 | 34.76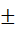2.04 | 25.53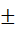2.83 |
| Jun | 26.69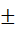2.61 | 29.77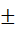3.31 | 30.3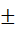3.39 | 28.98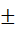2.76 | 33.92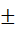2.58 | 35.56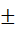2.71 | 26.28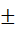2.82 |
| Jul | 33.23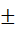2.07 | 35.23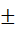2.94 | 36.33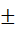2.53 | 34.45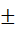2.35 | 36.43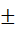1.89 | 40.99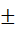2.45 | 32.65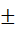2.23 |
| Aug | 30.26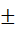2.09 | 31.93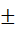2.55 | 32.65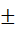2.33 | 30.88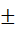1.77 | 32.02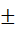2.09 | 37.11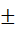2.95 | 29.56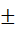1.68 |
| Sep | 27.27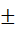1.85 | 29.56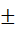2.01 | 29.81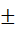2.14 | 28.49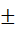1.54 | 29.88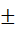1.74 | 34.02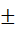2.13 | 26.67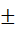2.07 |
| Oct | 21.21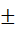2.07 | 22.29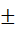2.1 | 22.83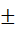1.95 | 21.86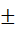1.64 | 22.93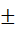1.36 | 24.63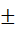1.8 | 20.38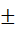1.77 |
| Nov | 18.58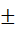1.81 | 18.75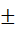2.19 | 19.74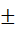1.88 | 17.79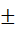1.85 | 19.43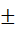1.72 | 20.74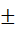2.07 | 15.54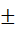2.25 |
| Dec | 10.25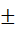1.27 | 11.46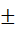1.25 | 11.47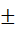1.12 | 10.61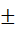1.35 | 11.98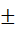0.83 | 12.24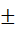0.96 | 8.82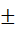2.33 |

Table B2. Land surface temperatures for different types of land cover in the nighttime. (Mean ± Std, ℃)

|  | Forest | Shrub land | Grassland | Wetland | Farmland | Bulit -up | Water |
| --- | --- | --- | --- | --- | --- | --- | --- |
| Jan | 3.21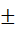1.10 | 2.28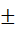2.06 | 2.27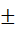1.42 | 2.96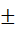1.97 | 0.66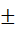1.24 | 2.11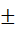1.36 | 3.66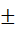2.73 |
| Feb | 4.61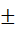1.01 | 3.90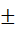1.60 | 3.29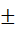1.36 | 3.82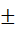1.85 | 1.59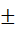1.25 | 2.84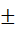1.09 | 5.77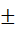2.12 |
| Mar | 3.42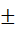1.01 | 5.601.97 | 3.491.21 | 5.271.64 | 2.311.23 | 3.991.12 | 7.842.22 |
| Apr | 10.961.07 | 12.781.63 | 11.271.28 | 13.271.56 | 10.820.99 | 11.991.04 | 15.211.74 |
| May | 15.521.27 | 17.642.18 | 16.141.41 | 18.091.73 | 15.741.18 | 17.061.16 | 19.921.60 |
| Jun | 16.511.49 | 19.351.61 | 17.331.62 | 19.621.57 | 17.171.18 | 18.561.24 | 21.151.49 |
| Jul | 24.241.56 | 27.121.33 | 25.721.63 | 27.421.55 | 26.320.80 | 27.381.08 | 28.691.38 |
| Aug | 22.181.53 | 25.430.96 | 23.861.50 | 25.401.20 | 24.340.84 | 25.280.91 | 26.391.03 |
| Sep | 15.712.16 | 18.871.92 | 16.831.95 | 18.922.01 | 16.871.27 | 18.021.62 | 21.182.00 |
| Oct | 12.531.33 | 14.901.48 | 12.771.29 | 14.561.54 | 11.751.10 | 13.241.06 | 16.191.60 |
| Nov | 7.911.52 | 9.662.13 | 7.251.70 | 9.072.00 | 5.421.62 | 7.401.47 | 11.752.21 |
| Dec | 1.861.26 | 3.632.02 | 1.181.38 | 2.892.28 | -0.15 | 1.181.23 | 4.852.40 |

1. ^a^ [↑](#footnote-ref-2)
